# Calcium imaging reveals glial involvement in transcranial direct current stimulation-induced plasticity in mouse brain

**DOI:** 10.1038/ncomms11100

**Published:** 2016-03-22

**Authors:** Hiromu Monai, Masamichi Ohkura, Mika Tanaka, Yuki Oe, Ayumu Konno, Hirokazu Hirai, Katsuhiko Mikoshiba, Shigeyoshi Itohara, Junichi Nakai, Youichi Iwai, Hajime Hirase

**Affiliations:** 1 RIKEN Brain Science Institute, 2-1 Wako, Saitama 351-0198, Japan; 2 Brain Science Institute, Saitama University, Saitama 338-8570, Japan; 3 Department of Neurophysiology and Neural Repair, Gunma University Graduate School of Medicine, Maebashi, Gunma 371-8511, Japan

## Abstract

Transcranical direct current stimulation (tDCS) is a treatment known to ameliorate various neurological conditions and enhance memory and cognition in humans. tDCS has gained traction for its potential therapeutic value; however, little is known about its mechanism of action. Using a transgenic mouse expressing G-CaMP7 in astrocytes and a subpopulation of excitatory neurons, we find that tDCS induces large-amplitude astrocytic Ca^2+^ surges across the entire cortex with no obvious changes in the local field potential. Moreover, sensory evoked cortical responses are enhanced after tDCS. These enhancements are dependent on the alpha-1 adrenergic receptor and are not observed in IP_3_R2 (inositol trisphosphate receptor type 2) knockout mice, in which astrocytic Ca^2+^ surges are absent. Together, we propose that tDCS changes the metaplasticity of the cortex through astrocytic Ca^2+^/IP_3_ signalling.

## Abstract (web summary)

While transcranical direct current stimulation (tDCS) is used in clinical setting, its cellular mechanism of action is unclear. Here, Hajime Hirase and colleagues visualize cellular response in mouse brain to tDCS and show robust astrocyte activation that coincide with plasticity changes.

## Graphical abstract

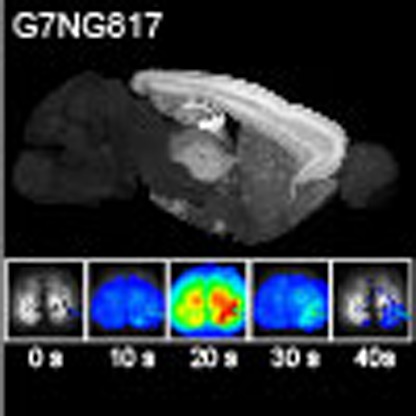

Transcranial direct current stimulation (tDCS) is a non-invasive brain stimulation procedure, in which weak d.c. current (typically 1 mA) is applied over cortical areas for 10–30 min through the skull. tDCS has been shown effective for alleviating neuropsychiatric and neurological conditions such as major depression in humans^1^. tDCS has also been demonstrated to enhance learning and memory formation^2^,^3^. Previous work demonstrated that tDCS applied over the motor cortex of human subjects increases the excitability of the motor cortex in a *N*-methyl-D-aspartate receptor (NMDAR)-dependent manner^4^. Application of d.c. over mouse motor cortical slice has demonstrated that DCS enhances synaptic response that depends on the NMDAR and brain-derived neurotrophic factor^5^. However, detailed cellular mechanisms for *in vivo* tDCS-induced plasticity remain largely unknown.

Multiple studies show crucial roles of astrocytes in NMDAR-dependent plasticity^6^,^7^,^8^,^9^, using rodent *in vitro* and *in vivo* preparations. These studies imply that astrocytes make gliotransmission to secrete signalling molecules to synapses. Several lines of evidence that gliotransmission occurs by intracellular Ca^2+^ elevation have been reported, although the exact mechanism of gliotransmission is still controversial^10^,^11^. As recent animal models of tDCS have successfully demonstrated enhancements in learning and synaptic plasticity^2^,^12^,^13^, we sought to address the dynamical activity of the brain during tDCS by Ca^2+^ imaging.

G-CaMP7 is a green fluorescent protein (GFP)-based Ca^2+^ indicator protein that was improved from the original G-CaMP^14^ by directed site mutagenesis to yield higher signal-to-noise ratio and faster response time, making it suitable for monitoring neuronal activity^15^. Here we introduce a transgenic mouse line ‘G7NG817' in which astrocytes and a subset of cortical neurons express G-CaMP7 permitting transcranial imaging, using a standard stereo fluorescence microscope. Surprisingly, we find that tDCS invokes large-amplitude and synchronized Ca^2+^ surges in astrocytes. This phenomenon lead to the hypothesis that tDCS-induced astrocytic activity affects the metaplasticity of the cortex in a similar manner to astrocytic modulation of synaptic plasticity by neuromodulators^16^.

## Results

### Functional mapping of the cortex using the G7NG817 mouse

**Figure 1:**
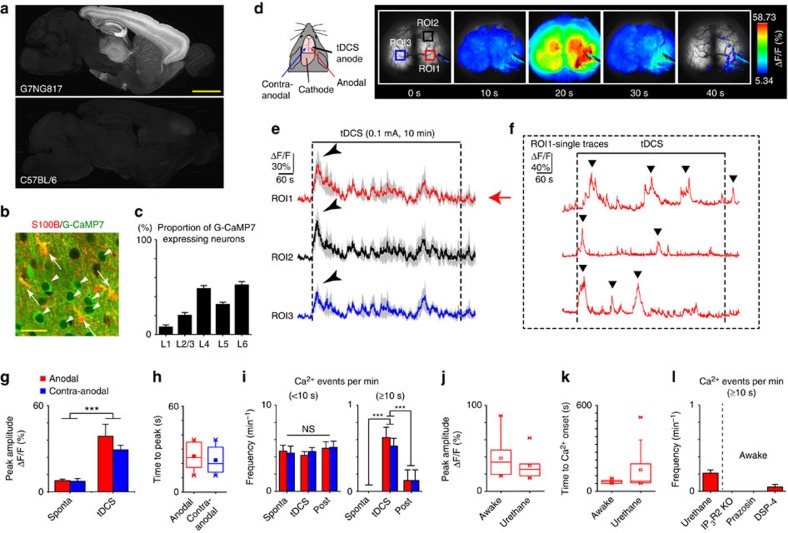
tDCS produces large and synchronized Ca^2+^ surges in the cortex as visualized with G7NG817 mice. (**a**) Sagittal brain section of the G7NG817 (upper panel) and C57BL/6 wild-type mice (lower panel). High expression of G-CaMP7 was seen in the cortex, hippocampus (particularly CA3), thalamus and striatum. Scale bar, 1 mm. (**b**) Immunostaining for G-CaMP7 (green) and astrocytes (S100B, red) shows a high degree of co-localization (arrows, co-stained astrocytes; arrowheads, G-CaMP7-positive neurons). Scale bar, 50 μm. (**c**) Bar graph that shows the layer-by-layer profile of G-CaMP7-positive cells in NeuN-labelled neurons (mean±s.e.m., *n*=9 slices from two mice) in the sensory cortex. (**d**) Sketch of the tDCS experiment configuration (left). The cathode is placed on the neck skin. On the right, the time course of an awake mouse transcranial imaging session during the initial 40 s of tDCS is displayed. The pseudocolour represents the range between the mean+1 s.d. and the peak value of the tDCS session. Three regions ROI1, 2 and 3 corresponds to the anodal, anterior and contra-anodal regions are illustrated for the following analyses. (**e**) Average peri-stimulus fluorescent intensity changes from multiple awake mice (*n*=8). Anodal (red), anterior (black) and contra-anodal (blue) regions are displayed. Shaded areas represent s.e.m. Arrowheads point to the initial large and long-lasting (≥10 s) Ca^2+^ surges triggered by tDCS. (**f**) Example traces of anodal cortical G-CaMP7 response during tDCS from three mice. Filled triangles (▾) indicate the occurrences of large and long-lasting (≥10 s) Ca^2+^ surges. (**g**) tDCS-induced Ca^2+^ surges have higher amplitudes than spontaneous Ca^2+^ events both on the anodal (red) and contra-anodal (blue) sites (anodal versus contra-anodal: *P*=0.30, sponta. versus tDCS: *P*=0.0001, two-way ANOVA). (**h**) Both anodal and contra-anodal sites have similar time to peak for the first Ca^2+^ surge since the onset of tDCS. (**i**) Long (≥10 s) Ca^2+^ surges are more frequent during tDCS (*P*=3.44E−7, two-way ANOVA), while shorter events occurred with a similar frequency (*P*=0.58, two-way ANOVA). Red and blue bars represent anodal and contra-anodal sites, respectively. (**j**) Distribution of the amplitudes of initial tDCS evoked Ca^2+^ surges in awake and urethane-anaesthetized mice. The amplitudes are similar between awake and urethane-anaesthetized mice. (**k**) Distribution of the onset of the initial tDCS evoked Ca^2+^ surge in awake and urethane-anaesthetized mice. The initial Ca^2+^ surge occurs within the first minute in the awake condition, whereas the onset time is more variable in urethane-anaesthetized mice. (**l**) tDCS-induced Ca^2+^ surges occur with a reduced frequency in urethane anesthesia compared with the awake condition (*n*=10 mice, cf. **i**). tDCS-induced Ca^2+^ surges do not occur in awake IP3R2 KO (IP_3_R2^−/−^;G7NG817^+/−^) mice (*n*=4). tDCS-induced Ca^2+^ surges are blocked by i.p. injection of prazosin (10 mg kg^−1^, 20 min before imaging) in G7NG817 mice (*n*=7) or in DSP-4-treated awake G7NG817 mice (*n*=7). ***P<0.0001. Error bars are defined as s.e.m.

The G7NG817 line was generated by a random insertion of a transgenic construct to express G-CaMP7 under the control of the GLT-1 promoter (see Methods). G-CaMP7 expression in the brain of the G7NG817 mouse was widespread and distinct from its background strain C57BL/6 (Fig. 1a). Particularly, high expression levels were observed in the cerebral cortex and hippocampus. Double immunostaining for S100B (astrocyte marker) and GFP (G-CaMP7 marker) shows that virtually all cortical astrocytes express G-CaMP7 (Fig. 1b; Supplementary Fig. 1A; 95.2%; *n*=3 mice), whereas other glia cell types show minimal expression (Supplementary Fig. 1C). In addition, neuronal expression of G-CaMP7 was observed (Supplementary Fig. 1D). The neuronal expression of G-CaMP7 in the cerebral cortex is not detectable in gamma-aminobutyric acid-ergic (GABAergic) cells (Supplementary Fig. 1E) and has a layer-dependent profile (Fig. 1c; Supplementary Fig. 1B). G-CaMP7 expression is also present in the processes of astrocytes and neurons, thus labelling the neurogliopil throughout the cortical depth. The high G-CaMP7 expression across the wide cortical area allowed transcranial imaging of neuronal and astrocytic Ca^2+^ activities of different cortical states (Supplementary Figs 2 and 3; Supplementary Video 1), as well as various sensory responses (Supplementary Figs 3 and 4; Supplementary Video 2).

### Activation of cortex-wide Ca^2+^ elevations caused by tDCS

Given the versatility of the G7NG817 mouse as a tool for monitoring cortical activity, we investigated brain activity during tDCS. We applied a 0.1 mA d.c. for 10 min between an anode at the cranial bone above the visual cortex (2 mm^2^ contact area) and a cathode attached to the neck of an awake mouse. To our surprise, a large Ca^2+^ elevation was observed within several seconds after the onset of tDCS (Fig. 1d; Supplementary Video 3). We find that the minimum current required to induce a Ca^2+^ surge is around 50 μA at the anode (Supplementary Figs 5 and 6). The peri-stimulus-averaged trace of the fluorescence signal in the vicinity of the anodal site (within 500 μm) from eight animals shows a distinct peak within several seconds (Fig. 1e,f). Similar peaks were seen at the ipsilateral anterior site (3 mm anterior from the anodal site) and the contralateral area (laterally symmetric to the anodal site).

We find that the tDCS-induced Ca^2+^ surges have larger amplitudes than spontaneous Ca^2+^ surges (sponta.) at both anodal and contra-anodal sites (Fig. 1g; sponta. versus tDCS Δ*F*/*F*; anodal: 7.70±1.1% versus 38.61±8.38%; contra-anodal: 7.27±1.94% versus 29.18±3.18%; anodal versus contra-anodal: *P*=0.30; sponta. versus tDCS: *P*=0.0001, two-way ANOVA). Moreover, tDCS-induced initial Ca^2+^ events appeared virtually simultaneously in the anodal, contra-anodal and distal regions of the cortex (Supplementary Video 3). Indeed, the time until the peak of a Ca^2+^ surge from the onset of the surge was similar between the two hemispheres (Fig. 1h). Remarkably, long-lasting Ca^2+^ surges (full-width half-maximum ≥10 s) occurred more frequently during tDCS (Fig. 1i, *P*=3.44E−7), while shorter events occurred with a similar frequency during tDCS (*P*=0.58). Furthermore, tDCS-induced Ca^2+^ surges in urethane-anaesthetized mice too with similar amplitudes (9 out of 10 mice), but the onset of the initial Ca^2+^ surge varied between mice (Fig. 1j,k) and the frequency of the long-lasting Ca^2+^ surges was reduced (Fig. 1l; *P*=0.0022). Notably, tDCS did not induce Ca^2+^ surges in awake G7NG817 mice that are deficient of inositol trisphosphate receptor type 2 (IP_3_R2)^17^ (Fig. 1l; Supplementary Fig. 7, Supplementary Video 4), the receptor responsible for large cytosolic Ca^2+^ elevations in astrocytes^18^. This result indicates that astrocytic Ca^2+^ elevation is likely the key contributor. Previous studies reported that alpha-1 adrenergic receptor (A1AR) signalling underlies Ca^2+^ elevations in cortical astrocytes in awake states^19^,^20^. In this regard, tDCS-induced cortical Ca^2+^ surges during awake states were blocked by the A1AR blocker prazosin or diminishment of noradrenergic innervation by N-(2-chloroethyl)-N-ethyl-2-bromobenzylamine (DSP-4) treatment^21^ (Fig. 1l, see Methods). The stimulation parameters were found to be sufficient to alleviate a mouse model of depression by chronic restraint stress (Supplementary Fig. 8). tDCS did not alleviate the depression-like behavior in mice with prazosin administration, DSP-4 treatment or IP_3_R2 deficiency. Moreover, we could not observe obvious astrocytic nor microglial reactivity after tDCS applied to awake mice (Supplementary Fig. 9), after 3 h.

### Astrocytes underlie tDCS-induced cortical Ca^2+^ surges

**Figure 2:**
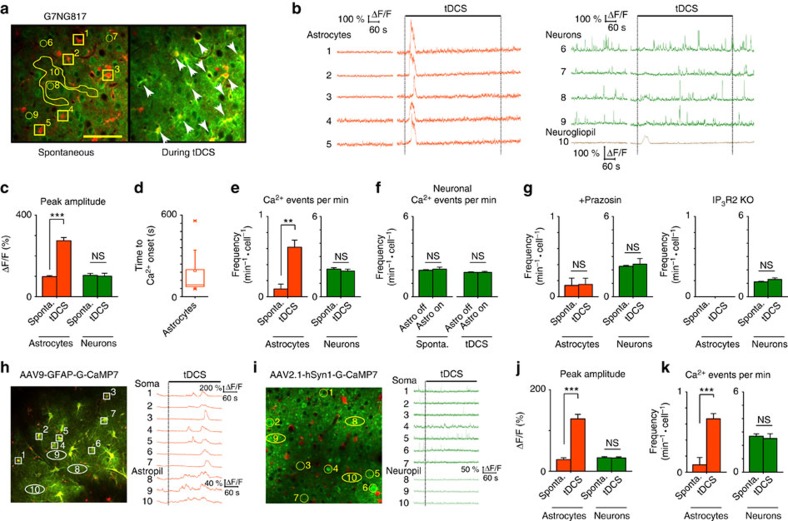
tDCS induces Ca^2+^ surges in layer 2/3 astrocytes, whereas neurons do not show obvious activity changes in urethane-anaesthetized mice. (**a**) Intracranial two-photon imaging of primary visual cortex layer 2/3 during tDCS. Astrocytes are labelled with SR101 (red). Arrows point to astrocytes that had Ca^2+^ elevations during tDCS. Numbers corresponds to the cells and neurogliopil region plotted in **b**. Scale bar, 100 μm. (**b**) Fluorescent intensity (Δ*F*/*F*) traces of astrocytes (orange), neurons (green) and neurogliopil (brown). (**c**) The tDCS-induced astrocytic G-CaMP7 signal amplitude (ΔF/F) is higher than that of spontaneous activity. There is no obvious change in neuronal signal amplitude (*n*=5, astrocyte: *P*=8.94E−34, two-sample *t*-test; neuron: *P*=0.15, two-sample *t*-test). (**d**) The variability of the initial tDCS-induced astrocytic Ca^2+^ surge timing. (**e**) The frequency of astrocytic Ca^2+^ surges increases during tDCS, while the neuronal Ca^2+^ event frequency does not show obvious changes (astrocytes: *n*=5 mice, *P*=0.0012, two-sample *t*-test; neuron: *n*=5 mice, *P*=0.42, two-sample *t*-test). (**f**) The mean frequency of neuronal Ca^2+^ events during (astro on) and outside (astro off) synchronized astrocytic Ca^2+^ surges (*n*=5, sponta: *P*=0.47, two-sample *t*-test, tDCS: *P*=0.12, two-sample *t*-test.). (**g**) tDCS-induced astrocytic Ca^2+^ elevation is blocked by topical application of prazosin (200 μM, 20 min before imaging, astrocytes: *n*=3, *P*=0.72, two-sample *t*-test; neuron: *P*=0.92, two-sample *t*-test) or in IP_3_R2 KO mice (*n*=3, neuron: *P*=0.16, two-sample *t*-test). (**h**,**i**) Cell-type-specific Ca^2+^ imaging of somata and processes during tDCS in urethane-anaesthetized mice. (**h**) Imaging of astrocytic Ca^2+^ dynamics. G-CaMP7 is expressed selectively in astrocytes by AAV9-GFAP-G-CaMP7. Both astrocytic somata and processes (=astropil) show long-lasting (≥10 s) Ca^2+^ surges during tDCS. (**i**) Imaging of neuronal Ca^2+^ dynamics. G-CaMP7 is expressed selectively in neurons by AAV2.1-hSyn1-G-CaMP7. Neither neuronal somata nor processes (=neuropil) shows obvious activity changes during tDCS. (j,k) Cell-type-specific imaging indicates that astrocytes, but not neurons, elicit elevations in the amplitude (**j**) and event frequency (**k**) of Ca^2+^ events (*n*=3, astrocytes: *P*=1.62E−15 and *P*=4.47E−4, two-sample *t*-test; neuron: *P*=0.86 and *P*=0.69, two-sample *t*-test). **P<0.001, ***P<0.0001. Error bars are defined as s.e.m.

To investigate the cellular mechanism of the tDCS-induced Ca^2+^ surges, two-photon imaging in layer 2/3 of the primary visual cortex was performed through a cranial window in urethane-anaesthetized G7NG817 mice. As demonstrated with transcranial imaging, synchronized and large-amplitude Ca^2+^ surges were evoked in a population of cells with tDCS (0.1 mA, 10 min; Fig. 2a). Notably, these cells were SR101-positive astrocytes (Fig. 2b). Further analysis revealed that the tDCS evoked astrocytic Ca^2+^ responses had significantly higher amplitudes than spontaneous events, whereas the neuronal Ca^2+^ events during tDCS had similar amplitudes as spontaneous events (Fig. 2c, comparisons between spontaneous versus tDCS periods for the cells that had at least one Ca^2+^ event during the respective period. Δ*F*/*F*: astrocyte: 98.44±4.81% versus 274.70±15.61%, 289 versus 174 cells in five mice, *P*=8.94E−34; neuron: 110.8±3.68% versus 118.67±4.03%, 283 versus 271 cells from five mice, *P*=0.15). Similar to the observations with transcranial imaging in urethane-anaesthetized mice, astrocytic Ca^2+^ surges were induced at variable delay from the onset of tDCS under urethane anaesthesia (Fig. 2d). Notably, astrocytic Ca^2+^ surges occurred nearly seven times more frequently during the course of tDCS (10 min) than during the baseline, while neuronal activity did not show obvious changes (Fig. 2e, sponta. versus tDCS, neuron: 2.07±0.10 min^−1^·per cell versus 1.92±0.14 min^−1^·per cell, *P*=0.42; astrocytes: 0.09±0.06 min^−1^·per cell versus 0.62±0.09 min^−1^·per cell, *P*=0.0012). Of note, while tDCS-induced Ca^2+^ surges in individual astrocytes were synchronized within several seconds, travelling wave propagation was not observed. Next, we compared the neuronal Ca^2+^ event frequencies in time windows without astrocytic Ca^2+^ surges (astro off) versus times with astrocytic Ca^2+^ surges (astro on) during control or tDCS periods. In this comparison, we did not see any significant changes in the neuronal Ca^2+^ event frequency (Fig. 2f).

Similar to the result that tDCS-induced Ca^2+^ surges were blocked by systemic administration of prazosin, local application of prazosin (200 μM topically applied on the craniotomy 20 min before imaging) blocked the tDCS-induced astrocytic Ca^2+^ surges, suggesting that noradrenergic activation of A1ARs is likely the key mechanism for the tDCS-induced Ca^2+^ surges (Fig. 2g). Furthermore, Ca^2+^ surges were not induced by tDCS in IP_3_R2 knockout (KO) mice (Fig. 2g and Supplementary Fig. 7), indicating that astrocytic GPCR activation is the prevalent mechanism of tDCS-induced Ca^2+^ surges.

While the Ca^2+^ signals from the somata of astrocytes and neurons are separable in G7NG817 by labelling glia with SR101, the neurogliopil signal is a mixed product of neurons and glia. To unambiguously investigate the contribution of cortical neurons and astrocytes to tDCS-induced Ca^2+^ surge, we expressed G-CaMP7 in neurons or astrocytes by cell-type-specific recombinant adeno-associated viruses (AAV2.1-hSyn1-G-CaMP7 and AAV9-hGFAP-G-CaMP7, respectively) in C57BL/6 mice (Fig. 2h,i). Using these preparations, neuron-specific neuropil and astrocyte-specific astropil were imaged. These experiments confirmed that astrocyte somata, but not neuronal somata, give rise to long-lasting (≥10 s) Ca^2+^ surges with tDCS. Moreover, the astropil showed Ca^2+^ responses after tDCS that co-occurred with the somatic Ca^2+^ surges (Fig. 2h), whereas the neuropil from local neurons did not respond to tDCS (Fig. 2i). The analysis of the cell-type-specific imaging shows that tDCS activates astrocytic, but not neuronal, Ca^2+^ elevations (Fig. 2j,k). Moreover, local field potential (LFP) patterns in the vicinity of the anode did not show obvious alternations with tDCS (Supplementary Fig. 10) in anaesthetized mice.

### tDCS-induced sensory plasticity depends on A1AR and IP_3_R2

**Figure 3:**
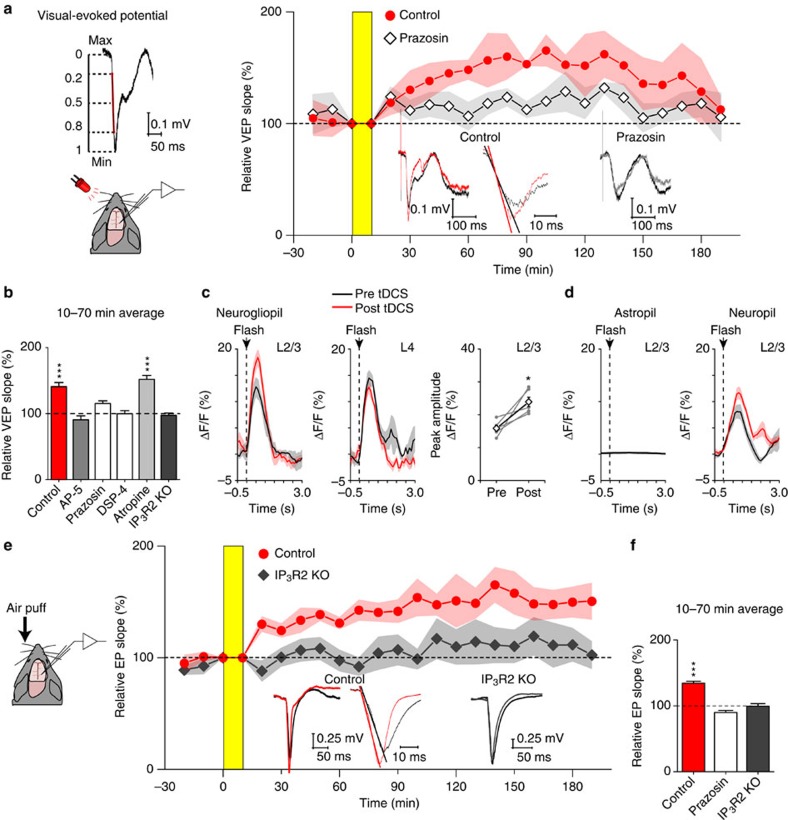
Sensory responses are enhanced by tDCS in a manner dependent on noradrenergic and astrocytic Ca^2+^/IP_3_ signalling in urethane-anaesthetized mice. (**a**) Schematics of visual flash evoked potential (VEP) measurement and evaluation of VEP slope. Example traces of averaged VEP recorded from the primary visual cortex in wild type (WT, left). The VEP slope is quantified for the interval covering 20–80% of the initial response (red). The VEP slope at the anodal side is enhanced after tDCS in naive (red, *n*=6), but not in mice with prazosin (grey, *n*=6). Yellow area represents tDCS application. Shaded areas represent s.e.m. Inset: examples of mean VEPs for a naive mouse (red) and a prazosin mouse (grey) at 70 min after the onset of tDCS are compared with pre-tDCS response (black). (**b**) tDCS-induced VEP slope enhancement is blocked by AP-5 (*n*=5, *P*=0.86), prazosin (*n*=6, *P*=0.26) and DSP-4 (*n*=6, *P*=0.26) treatment, but not by atropine (*n*=6, *P*=1.71E-8). Moreover, tDCS-induced VEP slope enhancement is absent in IP3R2 KO mice (*n*=12, *P*=1.0). Statistical significance was tested with one-way ANOVA followed by Tukey's post-hoc test. (**c**) Neurogliopil response to visual flash stimulation in G7NG817 mice is enhanced after tDCS in layer 2/3 (*P=0.010, *n*=4 mice), but not in layer 4. (**d**) Layer 2/3 neuropil Ca^2+^ response is significantly enhanced after tDCS (*n*=3, *P*=0.0062, paired *t*-test), whereas layer 2/3 astropil did not respond to visual flash stimulus (*n*=6). (**e**) tDCS-induced enhancement of sensory response in the whisker-barrel cortex system. Single air puffs are applied to whiskers contralateral to anodal and LFP recording side. Red and gray plots represent WT and IP_3_R2 KO mice, respectively. Yellow area represents tDCS. Inset: examples of mean VEPs for a WT mouse (red) and an IP_3_R2 KO mouse (gray), as in **a**. (**f**) tDCS enhances the whisker evoked LFP response in the barrel cortex of WT (*n*=7, *P*=8.08E-13) and blocked by topical application of prazosin (*n*=6, *P*=1.0). tDCS does not facilitate the whisker response in the barrel cortex of IP3R2 KO mice (*n*=8, *P*=1.0). Statistical significance was tested with one-way ANOVA followed by Tukey's post-hoc test. ***P<0.0001. Error bars are defined as s.e.m.

tDCS has been reported to have positive effects on memory and plasticity in animal models and in humans^3^,^4^,^12^. We sought to address the plastic effects of tDCS by investigating the visual evoked potential (VEP) in the primary visual cortex for an light-emitting diode flash stimulation. Urethane-anaesthetized C57BL/6 naive mice were used to record VEPs before and after tDCS (Fig. 3a). As a result, VEP slope increased by 50% after tDCS and remained potentiated for at least 2 h. This plasticity was blocked by the NMDAR antagonist AP-5, suggesting that glutamatergic synapses are involved (Fig. 3b). To investigate the contribution of A1ARs in the tDCS-induced plasticity, we repeated the experiment with topical application of prazosin (200 μM, applied before 20 min stimulus presentation). Remarkably, tDCS did not enhance the VEP in the prazosin condition (Fig. 3a,b) or in DSP-4-treated mice, whereas the muscarinic receptor antagonist atropine (2–3 mM) did not block the effect. Moreover, tDCS-induced VEP slope enhancements were not observed in IP_3_R2 KO mice (*n*=5 mice for AP-5, 12 mice for IP_3_R2 KO mice and 6 mice for all other groups). These results suggest an essential role of astrocytic Ca^2+^ elevations in the tDCS-induced enhancement of VEP. To examine the origin of LFP enhancement, neurogliopil imaging was performed with G7NG817 mice in layer 2/3 and 4 of the primary visual cortex. The neurogliopil response was enhanced in layer 2/3, but not in layer 4 (Fig. 3c). We next monitored visual flash responses of layer 2/3 astropil and neuropil by imaging after cell-type-specific expression of G-CaMP7 with AAV (Fig. 3d, *n*=6 mice for astropil expression by AAV9-hGFAP-G-CaMP7, *n*=3 mice for neuropil expression by AAV2.1-hSyn1-G-CaMP7). As a single visual flash does not result in Ca^2+^ elevation in astropil in layer 2/3 (Fig. 3d), the neurogliopil results in Fig. 3c likely reflect responses from neurons. Indeed, neuropil imaging confirmed that neuropil response in layer 2/3 was significantly enhanced after tDCS (Fig. 3d, *P*=0.0062).

The sensory response enhancement by tDCS was also seen in the whisker-barrel cortex system (*P*=8.08E−13, *n*=7, 6 and 8 mice for control, prazosin and IP_3_R2 KO experiments, respectively). Similar to the visual system, tDCS-induced plasticity was blocked by prazosin and was not present in IP_3_R2 KO mice (Fig. 3e,f).

**Figure 4:**
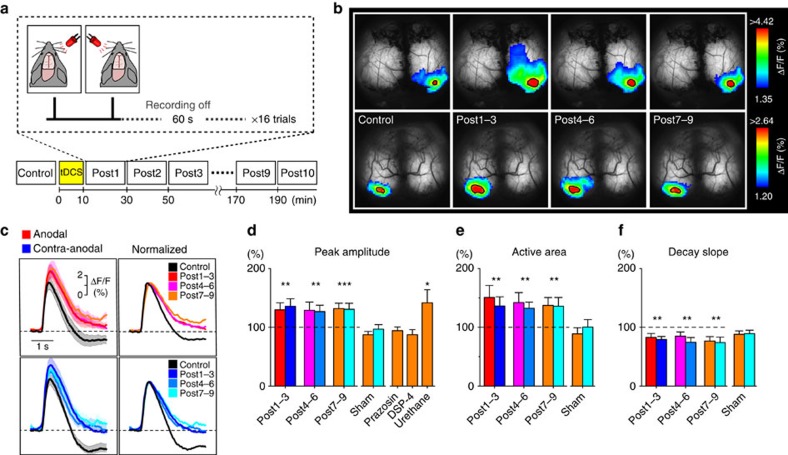
tDCS-induced enhancement of visual response is observed by transcranial Ca^2+^ imaging in awake and anaesthetized conditions. (**a**) Schematic diagram of the visual flash and transcranial imaging experiment. (**b**) Transcranial imaging of visual flash response. tDCS induces an enhancement of visual flash response in activation area and amplitude (single example). Left- and right-eye flash responses are plotted in upper and lower rows, respectively (ipsilateral responses are masked out). The colour range is between mean+1 s.d. and the peak value of the baseline visual evoked response. Areas exceeding 90% of the baseline visual evoked response (active areas) are demarcated by solid black borders. (**c**) Visual evoked Ca^2+^ response is enhanced after tDCS. In the left panel, mean fluorescence responses from G7NG817 mice (*n*=8) are plotted for the anodal (upper) and contra-anodal (lower) sites. Pre-tDCS control response is plotted in black and time periods after tDCS are in colour. Shaded areas represent s.e.m. In the right panel, these traces are normalized to the peak to show that the decay dynamics are altered after tDCS. (d–f) tDCS enhances amplitude (**d**), expands the active area (**e**) and attenuates the decay slope (**f**). Sham-operated control mice (*n*=7, *P*=0.51, two-way ANOVA) do not show significant changes. The tDCS-induced facilitation of visual evoked response is absent in prazosin-injected (i.p. 10 mg kg^−1^, *n*=7) or DSP-treated (*n*=6) awake mice, but observable in urethane-anaesthetized mice (*n*=3, *P*=0.022, one-way ANOVA). **P<0.01, ***P<0.001. Error bars are defined as s.e.m.

To examine the effect of tDCS in cortical plasticity in awake mice, we monitored the cortical response for a visual flash stimulation with transcranial imaging of G7NG817 mice. Visual flashes were presented to either eye (10 s apart) and the visual stimulation was repeated at an interval of 60 s before and after tDCS (Fig. 4a,b). We analysed the visual evoked responses obtained before and after tDCS (Fig. 4c). An area of 1.0 mm by 1.0 mm centred at the peak of the visual evoked response during the control period was analysed. This area corresponded to the primary visual cortex. As a result, the amplitudes of the visual evoked responses on the anodal side were enhanced after tDCS (Fig. 4d, anodal Δ*F*/*F*, post1–3, relative to control: 129.80±11.95%, *n*=8 mice, *P*=0.0004). Furthermore, the visual evoked active area (see Methods) expanded after tDCS and the expansion persisted for at least 3 h (Fig. 4e, anodal active area relative to control: 150.66±20.42%, *P*=0.0001). We also note that the decay slope of the Ca^2+^ transient became less steep (Fig. 4f, *P*=0.0004). Similar changes were observed in the contra-anodal sites. Notably, tDCS-induced plasticity was blocked by acute systemic prazosin administration or in mice treated with DSP-4, pointing to the involvement of noradrenergic activation (Fig. 4d). This tDCS-induced plasticity was also observed in urethane-anaesthetized animals (*n*=3, *P*=0.022), excluding potential effects of a startle response.

## Discussion

We demonstrate that the G7NG817 mouse yields high enough G-CaMP7 expression in astrocytes and neurons to permit transcranial functional imaging with a standard fluorescent stereo microscope. As the BAC-GLT-1 promoter is known to be active predominantly in astrocytes^22^, the strong expression of the transgene in cortical neurons in G7NG817 is ectopic. Nevertheless, the fortuitous combination of highly expressing neurons and astrocytes resulted in a transgenic mouse that allows transcranial fluorescence imaging for functional mapping of the cerebral cortex, even with heterozygous mice (Supplementary Fig. 11).

Although tDCS has been shown to have positive effects on memory and remedial effects on neuropsychiatric and neurological conditions in humans^1^,^23^, its mechanism of action is largely unknown. Using tDCS parameters commonly used for small rodent models^23^,^24^,^25^,^26^,^27^,^28^, we find that the activation of cortical neurons was undetectable by the analysis methods we used. This is reasonable, as the stimulus parameters used in this study are estimated to be close to the neuronal activation threshold reported in previous studies, which used a voltage-based approach^29^,^30^. Unexpectedly, tDCS-induced virtually synchronous astrocytic Ca^2+^ surges in wide-spread cortical areas.

We demonstrated enhancements of sensory evoked cortical responses after tDCS, consistent with an earlier study performed with the rabbit whisker system^12^. In addition, we show that the lack of the tDCS-induced plasticity in IP_3_R2 KO mice, suggesting the involvement of astrocytic Ca^2+^/IP_3_ signalling. Of note, recent reports have shown that astrocytic Ca^2+^/IP_3_ signalling plays a significant role in *in vivo* synaptic plasticity in the cortex and hippocampus^7^,^31^,^32^,^33^. Similar to a human study^4^, we showed that the tDCS-induced enhancement of sensory evoked LFP response is NMDAR dependent. As astrocyte Ca^2+^ levels are positively related to the extracellular level of the NMDAR co-agonist d-serine, tDCS-induced astrocytic Ca^2+^ elevations conceivably lead to NMDAR-dependent synaptic plasticity^6^,^7^,^9^,^34^,^35^.

A large part of the tDCS-induced plasticity was blocked by prazosin or DSP-4 treatment, indicating the involvement of noradrenergic activation of A1ARs, which transduce the G_q_ signalling cascade for production of IP_3_. Of note, a recent study by Panktratov and Lalo^35^ describes that noradrenaline application elevates extracellular d-serine and ATP levels, and lowers the threshold for the induction of LTP-like plasticity in mouse cortical slices. The same study also demonstrated little neuronal Ca^2+^ elevation after noradrenaline application, supporting our result that the tDCS-associated Ca^2+^ elevations are of astrocytic origin. Moreover, activation of A1ARs have recently been shown to be the prevalent mechanism of astrocytic Ca^2+^ elevation in awake mice^19^,^20^. The enhanced LFP response following tDCS is most likely due to a reflection of synaptic current enhancement, as rodent astrocytic membrane potential fluctuation is modest^36^. Our differentiation of neurogliopil signal into neuropil and astropil components confirmed that tDCS-induced plasticity occurs in neurons. Collectively, we have identified a major intermediate mechanism between the impact of the electrical field and neural plasticity mediated by astrocytic activity (Supplementary Fig. 12).

Potential mechanisms for this tDCS-induced noradrenergic drive include activation of the locus coeruleus and/or direct induction of transmitter release from noradrenergic axon terminals in the cortex. A simulation study predicts that axon terminals are more susceptible to polarization than somata by d.c. stimulation^37^. It is conceivable that the activation of neuromodulatory terminals are overlooked in our imaging experiments because the G-CaMP7 expression in non-glutamatergic neurons is low in G7NG817 and also single-axonal signals are not detected by LFP electrodes. The bias of noradrenergic activation over other neurotransmitters such as glutamate or acetylcholine is not clear. It is possible that glutamate and acetylcholine, if released in small amounts, undergo immediate transporter uptake or enzymatic breakdown before they reach to receptors. Future studies should identify the mechanism for the prevalent activation of A1ARs.

Finally, while our results suggest that the tDCS-induced parameters described in this study did not induce obvious glial inflammation, the safety standards of tDCS on humans^38^ should consider glial components in future.

## Methods

All experimental protocols were approved by the RIKEN Institutional Animal Care and Use Committee.

### Generation of transgenic mice

*G-CaMP7*-WPRE-polyA DNA^15^ was subcloned into a pCR-FRT-*Amp*-FRT plasmid^39^. A bacterial artificial chromosome (BAC) clone, RPCI-23-361H22 (BAC PAC Resources), containing the *GLT1* gene was modified by the Red/ET recombination system (Gene Bridges) to insert the PCR fragment of *G-CaMP7*-WPRE-polyA-FRT-*Amp*-FRT immediately downstream of the initiation codon. After selection of recombined colonies by ampicillin resistance, the *Amp* cassette was removed from the recombined BAC clones by introducing the Flp recombinase expression plasmid 706-FLP (Gene Bridges). The resulting BAC construct was amplified, purified with the Large-Construction kit (Qiagen) and digested with AscI. Correct modification of the BAC was verified by pulsed-field gel analysis of restriction digests and direct sequencing of the insert. The linearized BAC DNA was purified, adjusted to be ∼1 ng μl^−1^ in a microinjection buffer (10 mM Tris-Cl, 0.1 mM EDTA, 100 mM NaCl, pH 7.4), and individually injected into the pronuclei of 590 C57BL/6 J-fertilized embryos. As a result, 88 founders were born, of which 14 founders were positive for the transgene. Resulting founder mice were identified by PCR using the following primer pair: 5′- CGAGGCGCTAAAGGGCTTACC-3′ and 5′-GTACCGCCCTTGTACAGCTC-3′. Positive founder mice were crossed with C57BL/6 J mice to obtain germline transmission and the mouse lines were maintained on this genetic background. Despite the well-known-specific promoter activity of GLT-1 in astrocytes in the cerebral cortex^22^,^33^,^40^, we could not obtain any founders that had astrocyte-specific visible fluorescence expression levels with this construct. Rather, we obtained several lines in which visible amounts of fluorescence were seen in neurons of the cerebral cortex, hippocampus, striatum and other regions of the brain. Of these lines, line 817, which we term G7NG817, exhibited high fluorescence levels in the cerebral cortex. Homozygous transgenic mice were bred and used in the current study except for the experiments described in Supplementary Fig. 11, where heterozygous mice were used.

### Surgical procedures for virus innoculation

pAAV-hGFAP-G-CaMP7 was synthesized by modifying the pAAV-hSyn-EGFP vector (Addgene plasmid #50465, a gift from Bryan Roth) with a human GFAP promoter^41^ and G-CaMP7 complementary DNA. AAV9-hGFAP-G-CaMP7 was purified at a titre of 1.2 × 10^14^ vg ml^−1^. AAV2.1-hSyn1-G-CaMP7 (ref. 42) (7.1 × 10^13^ vg ml^−1^) is a gift from Masanori Matsuzaki (National Institute for Basic Biology, Japan). The viruses were diluted to 5–7 × 10^12^ vg ml^−1^ with phosphate-buffered saline (PBS) for microinjection. Mice were anaesthetized with isoflurane (1.5%) or ketamine+xylazine (intraperitoneal (i.p.), 56 and 8 mg kg^−1^, respectively), and fixed in a stereotaxic frame. A small craniotomy was made at the site of prospective imaging and a glass micropipette containing AAV was inserted to a depth of 250 μm below the surface of the cortex. Microinjection of 300 nl was made over 5 min using a Femtojet injector (Eppendorf). Imaging experiments were performed at least 2 weeks later.

### Surgical procedures for acute experiments

Male and female C57BL/6 wild type, G7NG817 and IP_3_R2 KO^17^ mice of postnatal 8–12-week old were used. The background strain of these mice is C57BL/6. Mice were housed under a 12 h/12 h light/dark cycle and raised in a group of up to five. Mice were anaesthetized with urethane (1.6 gkg^−1^) and the body temperature was maintained at 37 °C with a heating pad (BWT-100 A, Bio Research Center or TR-200, Fine Science Tools) during surgery and recording. After skull exposure, a metal frame was attached to the skull using a dental acrylic (Fuji LUTE BC, GC Corporation, Super Bond C&B, Sunmedical). For transcranial imaging, the skull was treated by a mixture of paraffin oil and glycerol (mixture composition) to increase transparency.

For two-photon imaging, a craniotomy (2 mm in diameter) was made above the visual cortex (anterior-posterior (AP) —2.0 mm, and mediolateral (ML) 3.0 mm). The dura mater was surgically removed. Sulforhodamine 101 (100 μM in PBS) was topically applied to label astrocytes and washed with N-2-hydroxyethylpiperazine-N′-2-ethanesulfonic acid (HEPES)-buffered artificial cerebrospinal fluid (ACSF) after 1 min. After the dye loading, the craniotomy was covered with agarose (1.5% w/v in ACSF) and gently sealed with a thin glass coverslip (3 × 3 mm, thickness: 0.12 mm, Matsunami Glass). The cranial window was secured by dental cement. For experiments involving LFP recording, a screw electrode (diameter, 0.7 mm; SUS-XM7, no. 00PH+14046, Matsumoto Industry) was implanted in the interparietal bone to serve as reference.

### Procedures for awake mouse recording

One to two weeks before the imaging session, mice were anaesthetized with a ketamine–xylazine cocktail (70 mg kg^−1^ ketamine and 10 mg kg^−1^ xylazine). After skull exposure, a stainless metal frame was attached to the skull using a dental acrylic. Following recovery, the mice were on a 24-h water-deprivation schedule and trained to be restrained under the microscope using a mechanical fixture that rigidly fixes the head frame once a day for 5–7 days. During training, mice have access to water once the head frame is fixed to the apparatus, thereby making an association between the head fixture and satiation of thirst.

### *In vivo* transcranial fluorescence imaging

Mice were fixed to a stereotaxic stage by clamping the head frame and placed under a fluorescence stereo microscope (MZ10F, Leica). The GFP3 filter set (excitation 470±20 nm, emission 525±25 nm, Leica) was used with the EL6000 light source (Leica). Images were acquired using the ORCA-Flash 2.0 CMOS camera (Hamamatsu Photonics) using HC Image software (Hamamatsu Photonics). The HC Image software also controlled a shutter unit to illuminate the skull only during imaging. Images were acquired with a size of 512 × 512 pixels and 16 bit resolution. For sensory stimulation experiments (Fig. 4; Supplementary Figs 3, 4 and 11), images were acquired with a 30-Hz frame rate. For tDCS experiments (Fig. 1; Supplementary Figs 5–7B), images were acquired at 10 Hz. For displaying purposes, the Δ*F*/*F* signal is thresholded at mean+s.d. for Figs 1d and 4b; Supplementary Fig. 3A, B and 4. Pseudocolouring was done using Origin 9.0 (Origin lab).

### *In vivo* two-photon imaging

Two-photon imaging was performed with urethane-anaesthetized adult mice (as above), using a resonant scanner-based B-Scope (Thorlabs) with a Chameleon Vision 2 laser (Coherent, wavelength 920 nm) and an Olympus objective lens (XLPlan N × 25). The B-Scope is equipped with a reverse dichroic mirror (ZT405/488/561/680-1100rpc, Chroma) and the emission light was separated by using a dichroic mirror (FF562-Di03, Semrock), with band-pass filters FF03-525/50 and FF01-607/70 (both from Semrock) for the green and red channels, respectively. Images were acquired using the ThorImage software with a frame rate of 30 Hz.

### Sensory stimulation

Sensory stimulation was presented to mice as follows. Visual flash stimulation: a flash of light from a red light-emitting diode (UR5365S, 20 mA, Stanley Electric) was presented to the right eye and then to the left eye 10 s later in darkness. This sequence was repeated every minute for 20 min. tDCS (see below) was applied 1 min after the visual flash stimulus. No visual flash stimulus was applied during tDCS. The same visual flash stimuli were presented 1 min after tDCS. For VEP plasticity experiments, the flash signal was presented to the left eye at an interval of 30 s in a room light condition. Whisker stimulation: individual whiskers were deflected at a frequency of 10 Hz for 5 s by a custom device made out of an audio speaker (Supplementary Fig. 4A). For whisker evoked response plasticity experiments, single air puffs were presented to the left whisker pad at an interval of 30 s (Fig. 3e,f). Auditory stimulation: 5-kHz pure tone for 500 ms (76 dB) was presented over a speaker using a custom LabView program (Supplementary Fig. 4C). Tail pinch: tail pinch was manually applied in anaesthetized mice via blunt tongs for several seconds.

### Transcranial d.c. stimulation

An anode was placed on a conductive (PBS with 1.5% agarose) gel interface spreading over a 2 mm^2^ area above the primary visual cortex. A cathode was inserted to the neck muscle for experiments with anaesthetized mice. For experiments with unanaesthetized mice, a cathode was placed on the neck skin. D.c. was applied from the anode to the cathode with a stimulus isolator (ISO-Flex, AMPI) or a custom-made isolated constant-current supply, powered by a 9-V battery. Unless otherwise noted, current intensity and duration for tDCS are set to be 0.1 mA and 10 min, respectively. The potential difference between the anode and cathode was typically around 0.5 V.

### Local field potential recording

Extracellular recordings were performed with an ELC-03XS amplifier (NPI electronic). A glass micropipette (2-μm tip diameter, 1B150F-4, World Precision Instruments) was filled with HEPES-ACSF (pH 7.4) and placed to an electrode holder with a headstage preamplifier. The headstage is then mounted to a remote-controlled micromanipultor (Sensapix). Under a stereo microscope, the glass micropipette was inserted to the primary visual cortex (250 μm below the pia) at a 30° insertion angle. Recording sessions started 1–2 h after insertion of the electrode for the stabilization of evoked responses^43^. After amplification (2000 ×, 0.1 Hz to 3 kHz), the signal was digitized at 20 kHz and stored on a hard drive using a LabVIEW-based data acquisition system. The field potential experiments have been performed in a room light condition.

### Drug application

For pharmacological experiments with cranial window (that is, two-photon imaging or LFP recording), reagents were dissolved in HEPES-ACSF (in mM:125 NaCl, 5 KCl, 10 glucose, 10 HEPES, 2 CaCl_2_ and 2 MgSO_4_) and applied onto the brain surface from 20 min immediately preceding imaging and the solution was kept in the cranial window until the end of the experiment. The concentrations of the applied drugs were: atropine sulfate monohydrate (2–3 mM, catalogue no. 015-04853, Wako Pure Chemical Industries)^7^, prazosin hydrochloride (200 μM, catalogue no. P7791-50MG, Sigma–Addrich)^19^ and AP-5 (50 μM, Tocris).

For i.p. administration of prazosin performed in transcranial imaging experiments, injection was made at a dosage of 10 mg kg^−1^ (1% w/v, dissolved in 0.9% NaCl). DSP-4 treatment was performed by a single i.p. injection of DSP-4 (50 mg kg^−1^, 12.5% w/v, dissolved in 0.9% NaCl) 7 days before the imaging experiment.

### Behavioural test

Chronic restraint stress and tail suspension test (TST) were performed as described in previous papers^44^,^45^. In brief, metal frame-attached mice were restricted to water access and socially isolated for 12 days (Supplementary Fig. 8A). Furthermore, each mouse was subjected to 8–9 h of chronic restraint stress for 5 consecutive days before behavioural test using a plastic cylinder with a radius of 8 cm and a height of 8 cm. After each session of restraint, animals were returned to their home cage with free access to food. For the behavioural test, all mice were exposed to a 9-min TST and the immobility time was counted (Supplementary Fig. 8B). Each mouse was individually suspended by their tail from a metal bar fixed 60 cm above the surface of a table with a soft vinyl tape. The test sessions were recorded by a video camera. The subjects were split into five groups: sham (*n*=10), tDCS (*n*=10), prazosin+tDCS (*n*=10), DSP-4+tDCS (*n*=9) and IP3R2 KO+tDCS (*n*=9). The protocols of tDCS and drug application were the same as described above.

The repeated TSTs were performed in six successive trials (Supplementary Fig. 8A). TST1: 5 days before tDCS as control; TST2: after experienced 5-day chronic restraint stress; TST3: 180 min after tDCS; TST4: 1 day after tDCS; TST5: 3 days after tDCS; and TST6: 1 week after tDCS.

### Histology

Mouse brains were perfusion-fixed with 4.0% paraformaldehyde (pH 7.4 in 0.1 M phosphate buffer). Following overnight postfixation in the same fixative, coronal slices (60 μm) were prepared using a microslicer (PRO 7, Dosaka). For GFP, NeuN, GFAP and IBA-1 staining, sections were incubated with primary antibodies (1:2,000 for anti-GFP, NeuN, GFAP and IBA-1 antibodies, Tris-buffered saline (TBS) with 0.1% Triton X-1000) overnight. For S100B staining, sections were first treated with 10% normal goat serum in 0.1% Triton X-100 TBS for 1 h, and then incubated with anti-S100B antibody (1:1,000) in 10% normal goat serum (in 0.1% Triton X-100 TBS) overnight. For GAD67 staining, we blocked the sections for 1 h using the Mouse on Mouse Polymer IHC kit (Abcam), and then incubated with anti-GAD67 antibody (1:500) in 0.1% Triton X-100 TBS for overnight. The sections were subsequently washed in phosphate buffer and incubated with Alexa 488- and 594-conjugated secondary antibodies (Invitrogen) for 2 h for fluorescent labelling. Immunolabeled sections were examined using a FV1000 confocal microscope (Olympus). Confocal images were taken with a 60 × oil immersion lens (UPlanSApo, numerical aperture (NA)=1.35). For Supplementary Fig. 9, the images were acquired with a × 20 objective (UPLanSApo, NA=0.75) with a *z*-step size of 2 μm and a × 60 water immersion lens (UPlanSApo, NA=1.2) with a *z*-step size of 0.5 μm, respectively. Primary antibodies were obtained from the following sources: GFP^46^, NeuN (Chemicon, MAB377), S100B (Sigma, S2532), GAD67 (Millipore, MAB5406), GFAP(DAKO, Z0334) and IBA-1 (Wako, 019-19741).

### Data analysis

*Extracellular field recording*. For spectral power analyses (Supplementary Fig. 10), the power spectral density was estimated by the Welch periodogram method using a Hamming window (window size=0.82 s; overlap=0.41 s). Recordings within 10 s from the onset or offset of tDCS were excluded from the analysis. The slope of evoked visual field response was calculated as follows. First, the initial deflection of the LFP response was isolated. Next, the region for slope calculation was defined as the interval within 20–80% of the peak-to-peak amplitude of the negative deflection 7 (Fig. 3a). The slope was computed by linear regression of the selected region.

*Transcranial imaging*. The original 512 × 512 pixel images were reduced to 64 × 64 pixels by binning. For sensory stimulus response imaging (Fig. 4; Supplementary Figs 2,3A,4 and 11), Δ*F*/*F* was calculated by taking the baseline *F* as the signal 500 ms before the stimulus presentation. For tail pinch or tDCS experiments (Figs 1 and 2; Supplementary Figs 3 and 7), the baseline *F* is defined as the mean intensity of the 50 s period ending 10 s before tail pinch or tDCS. For the analysis of the spontaneous slow oscillations (Supplementary Figs 2 and 6), the baseline *F* is defined as the mean intensity for the entire imaging period. Cross correlation was computed with the *Z* scores of the mean intensities of the region of interests (ROIs), so that the result is the Pearson correlation coefficient. Ca^2+^ events are defined as the events that had Δ*F*/*F* larger than the mean+1 s.d. for longer than 1 s and classified into two classes: those which had Δ*F*/*F* larger than mean +1 s.d. for shorter than 10 s (<10 s) and others that lasted longer (≥ 0 s; Fig. 1i,l; and Supplementary Fig. 11C). Time to Ca^2+^ onset is defined as the time where Δ*F*/*F* exceeded the mean+3 s.d. since the start of tDCS (Fig. 1h; Supplementary Fig. 11B). For the analysis of visual evoked response area, the active area is defined as the region that exceeds 90% of the mean peak response during the control period (Fig. 4b,e; and Supplementary Fig. 11F). To compute the decay slope, the mean visual evoked responses for respective periods were normalized to their peak value (Fig. 4f). The slope was computed similarly to VEP (Fig. 3a). All data are expressed as mean±s.e.m.

*Two-photon imaging*. The peak amplitude and duration statistics were made on cells that had at least one Ca^2+^ elevation during the course of 600 s imaging (Fig. 2c,d,j; Supplementary Fig. 3D). For each ROI, the baseline mean and s.d. of the signal were calculated for the first 60 s. The relative fluorescence change Δ*F*/*F* was computed, where the Δ*F* is the difference from the baseline mean and *F* is the baseline mean. Signal fluctuations in an ROI larger than the mean+3 s.d. were considered to be Ca^2+^ events (Fig. 2e,g,k; and Supplementary Fig. 7D). The duration is computed as the full-width half-maximum of the Ca^2+^ event (Supplementary Fig. 3E). For tail pinch experiments, the ‘during tail pinch' period is defined as the 100-s period starting from the onset of tail pinch. For tDCS experiments, the ‘tDCS' period is defined as the entire period of tDCS (that is, 600 s). All data are expressed as mean±s.e.m. Time to Ca^2+^ onset is defined as the time where Δ*F*/*F* exceeded the mean+3 s.d. since the start of tDCS. Ca^2+^ events for individual cells were detected with a threshold of the mean+3 s.d. Ca^2+^ events of astrocytes in G7NG817 mice were detected after applying a low-pass filter (cutoff frequency 0.5) to exclude possible neuronal signals from the contiguous neurogliopil (Fig. 2e,g,k). Ca^2+^ events of neurons in G7NG817 mice were detected after applying a band-pass filter (bandpass: 0.5–3 Hz) to exclude possible astrocytic signals from the contiguous neurogliopil (Fig. 2e–g,k). For the analysis of astrocyte-Ca^2+^ on versus off period (Fig. 2f), the ‘on' period is defined as the period where at least one astrocyte is detected for a Ca^2+^ event.

### Statistics

For comparisons of two sample means (Fig. 2c,e–g; Supplementary Figs 3C,E,F,7D), two-sample *t*-tests and paired *t*-tests (Fig. 3d) were performed using ORIGIN (OriginLab). For comparisons of multiple groups, statistical tests based on analysis of variance (ANOVA) are performed followed by Tukey s post-hoc tests. Two-way (Fig. 1g,i, and Fig. 4d-f) or one-way (Supplementary Fig. 10B) repeated measures ANOVA was computed using R (http://www.r-project.org) with a software add-on for ANOVA (ANOVAKUN, http://riseki.php.xdomain.jp/). One-way ANOVA was computed using ORIGIN (Fig. 1l and Fig. 3b,f; and Supplementary Fig. 8B). Unless otherwise noted, mean values are presented with the s.e.m.

## Additional information

**How to cite this article:** Monai, H. *et al*. Calcium imaging reveals glial involvement in transcranial direct current stimulation-induced plasticity in mouse brain. *Nat. Commun.* 7:11100 doi: 10.1038/ncomms11100 (2016).

## Supplementary Material

Supplementary Information Supplementary Figures 1-12

Supplementary Movie 1 Transcranially observed slow oscillations in anesthetized G7NG817 mouse. Urethane dosage: 1.7 g/kg, body temperature: 37.0{degree sign}C, the same mouse as presented in Supplementary Figure 2A. Acquisition and replay rate: 100 Hz

Supplementary Movie 2 Cortex-wide Ca2+ elevation by tail pinch in anesthetized G7NG817 mouse. Urethane dosage: 1.7 g/kg, body temperature: 37.0{degree sign}C, the same mouse as presented in Supplementary Figure 3B Acquisition and replay rate: 100 Hz

Supplementary Movie 3 tDCS-induced cortical Ca2+ surges in a unanesthetized mouse (as presented in Figure 1D). Acquisition rate: 10 Hz; replay rate: 30 Hz

Supplementary Movie 4 Lack of tDCS-induced cortical Ca2+ surges in IP3R2-/-;G7NG817+/- mouse (as presented in Supplementary Figure 7B) Acquisition rate: 10 Hz; replay rate: 30 Hz
