# Supplementary material for: Calcium imaging reveals glial involvement in transcranial direct current stimulation-induced plasticity in mouse brain
Source: Nat Commun. 2016 Mar 22;7:11100. doi: 10.1038/ncomms11100 (PMC4804173; doi:10.1038/ncomms11100)
Supplement: Supplementary Information — Supplementary Figures 1-12 [file ncomms11100-s1.pdf]

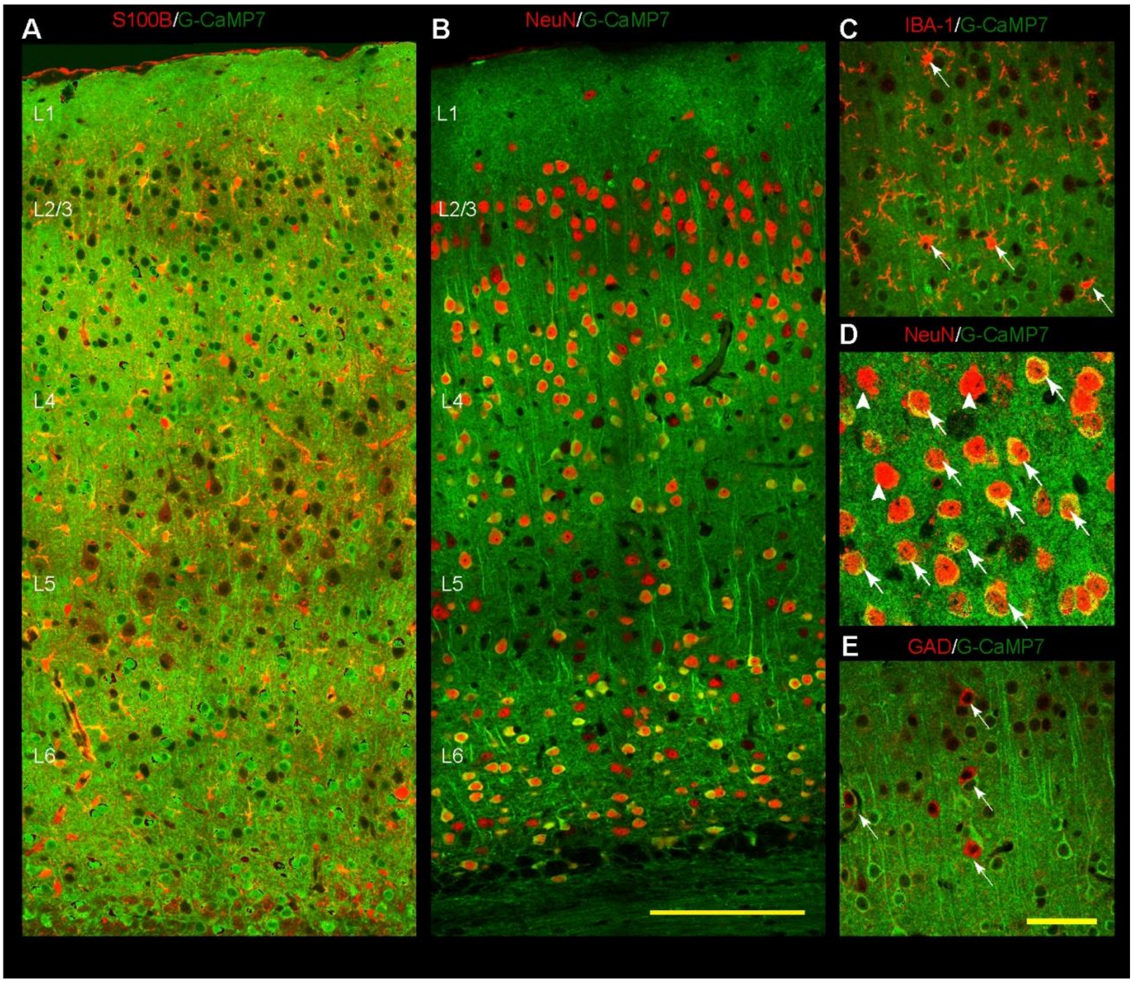

Supplementary Figure 1. Anatomical characterization of G-CaMP7 expression in the cerebral cortex of G7NG817

The primary somatosensory cortex of G7NG817 is immunostained with S100B antibody (A) and NeuN antibody (B, D) to label astrocytes and neurons, respectively (red). G-CaMP7 expression is enhanced with GFP immunostaining (green). G-CaMP7 expression is minimal in microglia (C, IBA-1 staining) or in GABAergic cells (E, GAD67 staining) in the cortical gray matter.

Scale bars: A, B 200 µm; C-E 50 µm

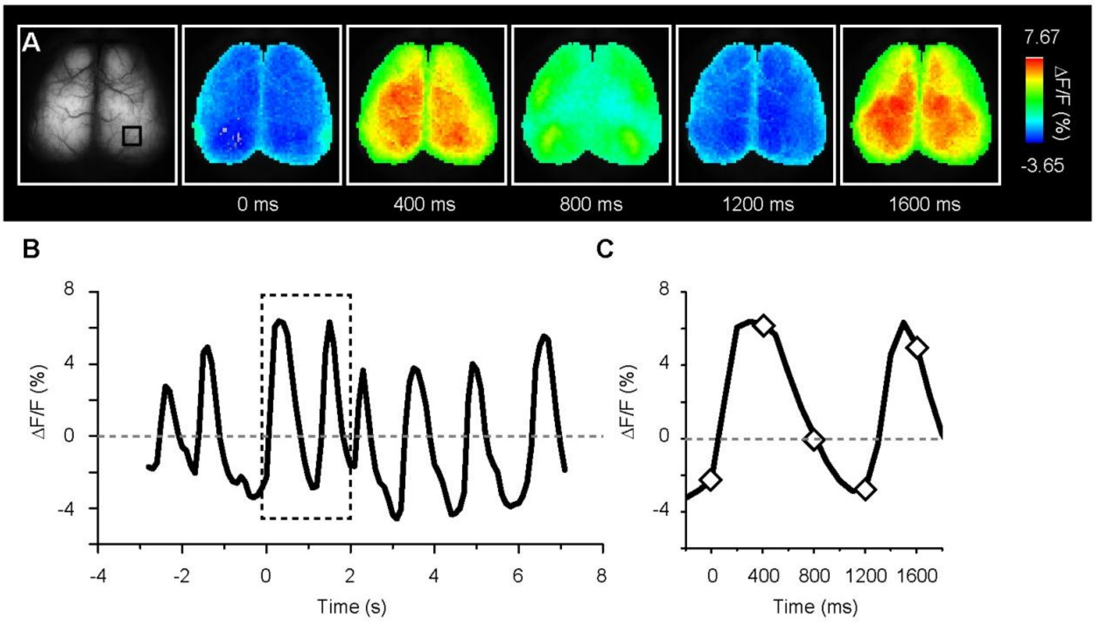

Supplementary Figure 2. Transcranial imaging of the slow oscillations during deep anesthesia in G7NG817

Transcranial imaging on a urethane anesthetized G7NG817 mouse displays large-amplitude and synchronized slow oscillations (A). The normalized fluorescence intensity (ΔF/F) from the visual cortex (A, black square) is plotted in b. The frequency of the oscillations ranges from 0.5 to 2 Hz, consistent with the LFP slow oscillations reported in urethane-anesthetized rodents. The images are taken at a frame rate of 10 Hz. The dotted area in B. is magnified in C. The diamond symbols represent the time points for the images displayed in A.

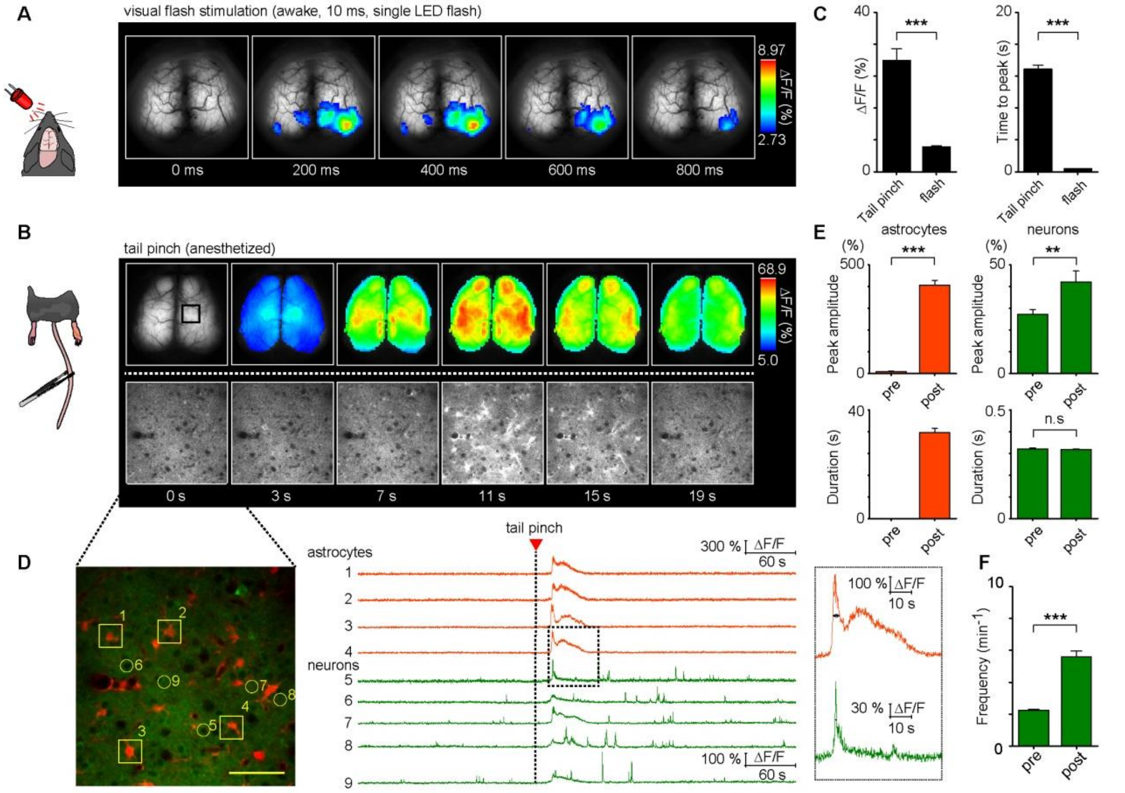

Supplementary Figure 3. Visual and tail-pinch responses of the cerebral cortex using G7NG817

A. Visualization of cortical dynamics in response to visual flash stimulation presented to the left eye of an awake mouse. Pseudocoloring is superimposed on the real images. The color range shown is between the mean + 1SD and peak values of the baseline visual evoked response.

B. Transcranial imaging of the entire dorsal cortical area to visualize the G-CaMP7 response to tail pinch in anesthetized animals (upper panel). Pseudocoloring is superimposed on the real images. The color range shown is between the mean + 1SD and peak values of the baseline visual evoked response. Note that the response amplitude is tenfold higher than those by flash visual stimulations and the time course is an order of magnitude slower. Two-photon imaging of the layer 2/3 of the sensory cortex during tail-pinch in a different mouse (lower panel).

C. Bar graphs for the comparisons of fluorescence signal intensity (left) and time course (right) between tail pinch and visual flash stimulations.

D. Astrocytic and neuronal Ca^{2+} activities were separated by the SR101 red fluorescent dye that loads selectively into glial cells. SR101-loaded astrocytes (orange) and neurons are marked with squares and circles, respectively (left) and their fluorescent signals are plotted. Red arrowhead indicates the timing of tail pinch. Note that the ΔF/F scale bars are 300 % and 100% for astrocytic and neuronal traces, respectively. The dotted region is magnified on the right panel to show the signal time course of the astrocyte and the neuron. Scale bar: 50 µm.

E. Bar graphs show that the tail-pinch induces large and long-lasting G-CaMP7 signal (ΔF/F) in astrocytes. Note that the average Ca^{2+} signal amplitude in neurons was also increased by tail pinch, possibly reflecting burst-like firing. pre: 20 s period before tail pinch. post: 20 s period after tail pinch.

F. The frequency of the neuronal Ca^{2+} events increases in the post-tail pinch period than the pre-tail pinch period.

*** $p < 0.001$, ** $p < 0.01$

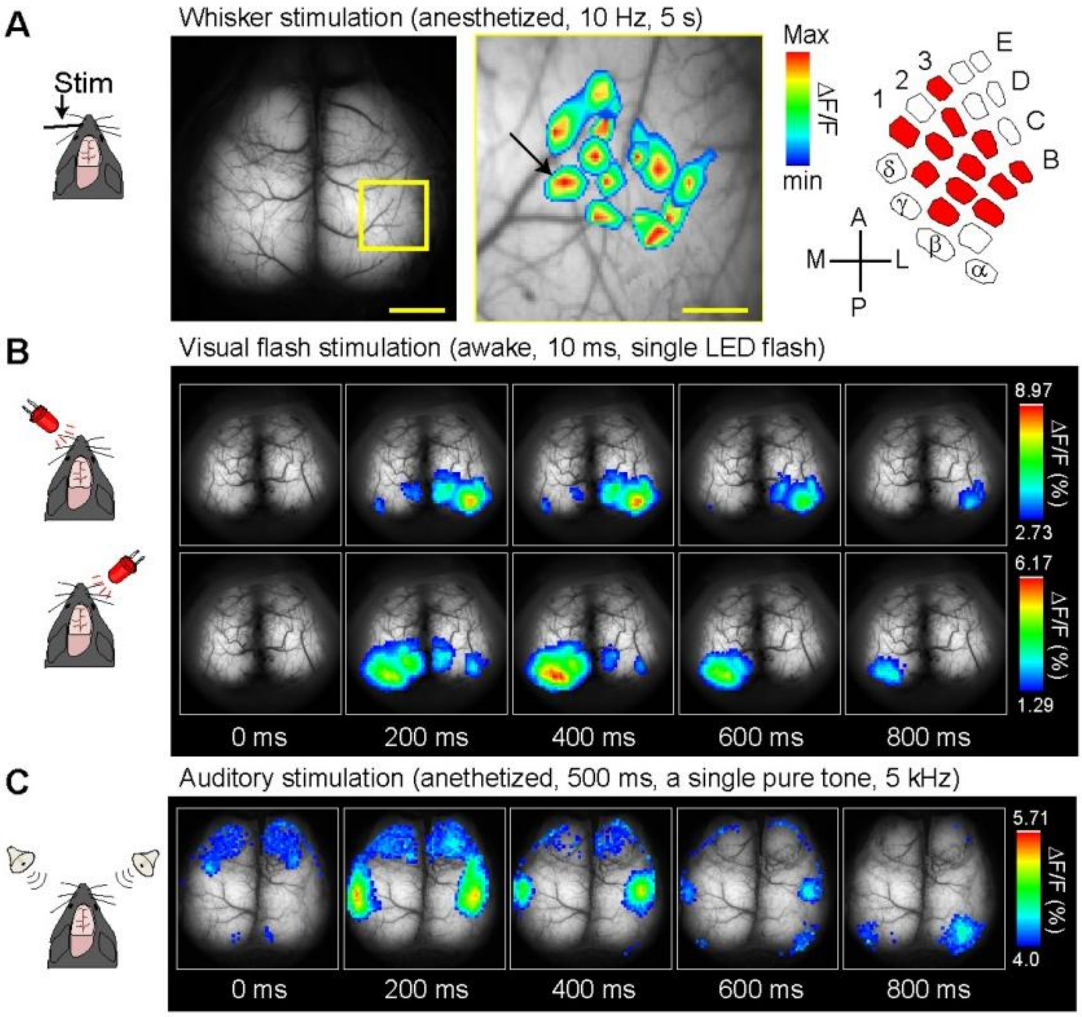

Supplementary Figure 4. Transcranial functional mapping of the cerebral cortex using G7NG817
A. To demonstrate the utility of G7NG817 for functional mapping, individual whiskers were deflected while transcranial imaging was made over the barrel cortex. The barrel area (yellow square) was imaged while individual whiskers were separately deflected at a frequency of 10 Hz for 5 s in an anesthetized G7NG817 mouse. The mean responses for individual whiskers (16 repetitions) are overlaid in the middle panel. The peak response for D1 whisker stimulation (arrow) is ΔF/F: 3.02 %. The stimulated whiskers and the corresponding barrel structure are shown in the right panel.
B. Functional mapping of other sensory modalities was also demonstrated in awake and head-restraint conditions. For instance, visual flash stimulation (10 ms) presented to either eye resulted in an activation of the contralateral visual cortex (average of 16 responses). The same mouse as in Supplementary Fig 3A is presented.
C. Visualization of cortical dynamics in response to pure tone presentation (5 kHz pure tone for 500 ms) to an anesthetized mouse. The primary auditory cortex is activated (average of 16 trials).
Scale bars: a, 1 mm, 250 µm.

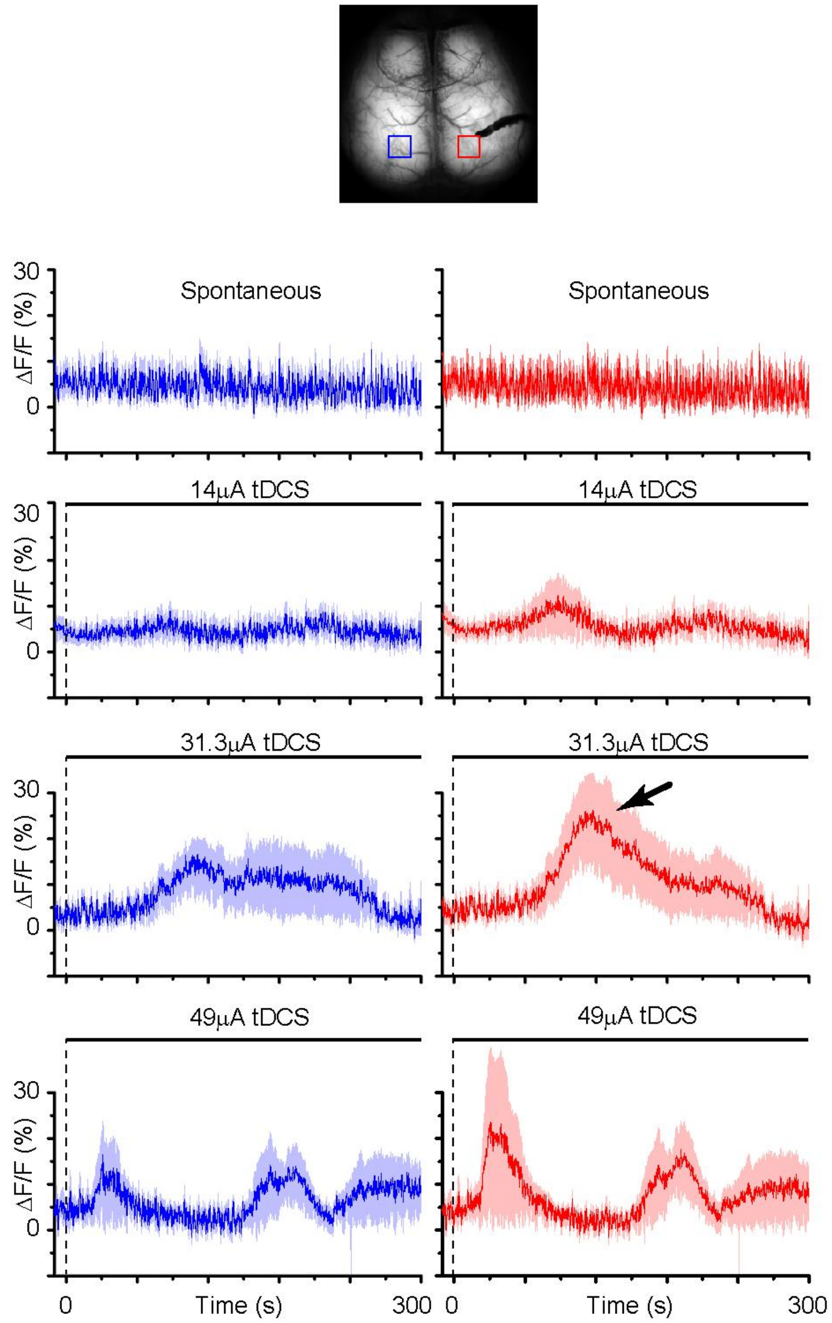

Supplementary Figure 5. Determination of the tDCS threshold for cortical Ca^{2+} surges

To determine the minimum current necessary to evoke Ca^{2+} surges by tDCS, the stimulus intensity was increased in steps. We find that 30-40 µA is sufficient to induce Ca^{2+} surges (arrow) in both hemispheres (N = 4 out of 5 mice).

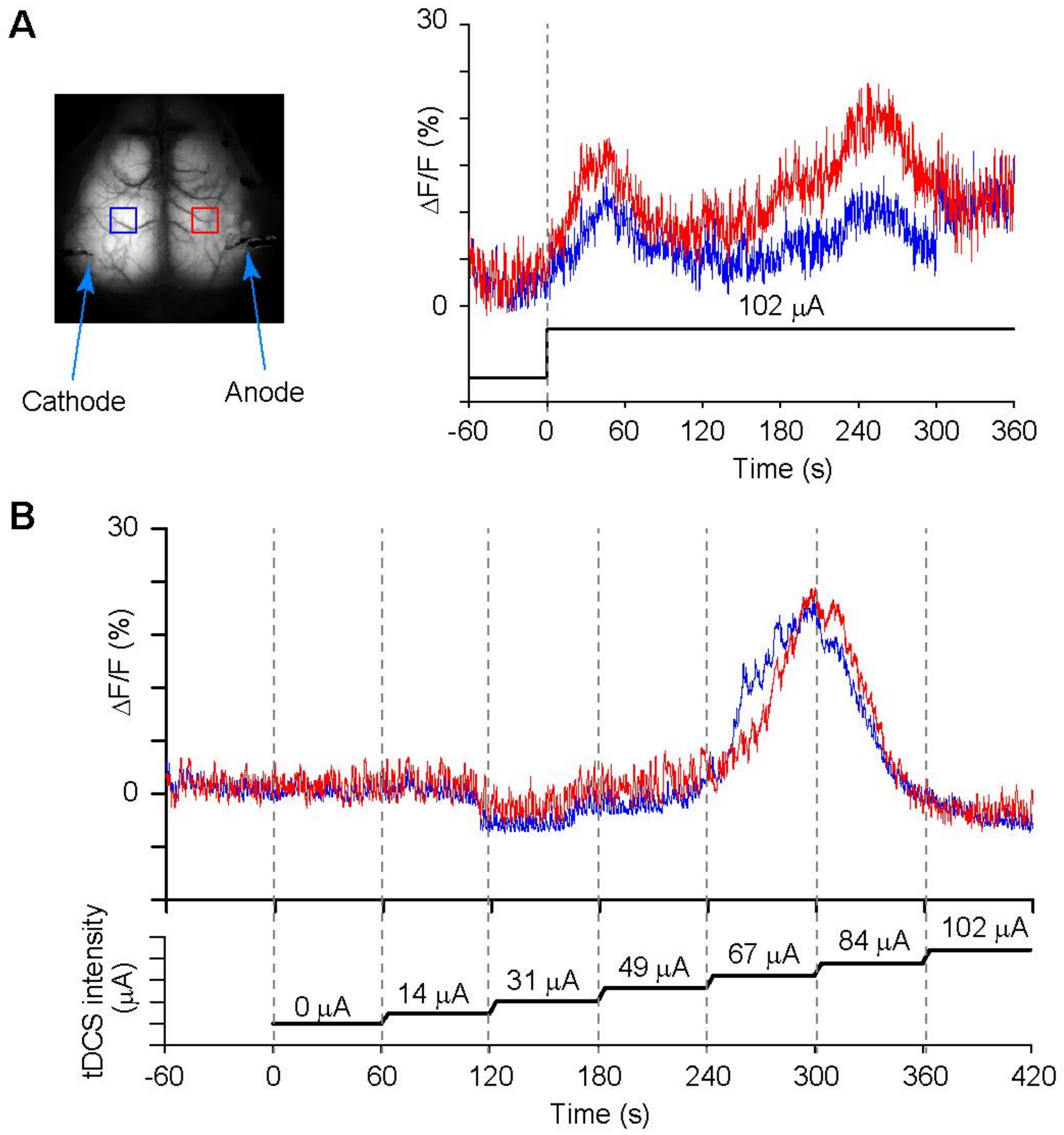

Supplementary Figure 6. Induction of Ca^{2+} surges by interhemispheric tDCS

A. To exclude the possibility that peripheral nerve stimulation by the cathode causes the activation of cortex-wide Ca^{2+} surges, we placed the anode and cathode on the skull. The interhemispheric tDCS also induced Ca^{2+} surges with a current intensity of 102 μA.

B. The tDCS current was gradually increased to investigate if the instantaneous current increase is critical in inducing a cortical Ca^{2+} surge. In this example, a Ca^{2+} surge was induced as the tDCS current exceed 50 μA.

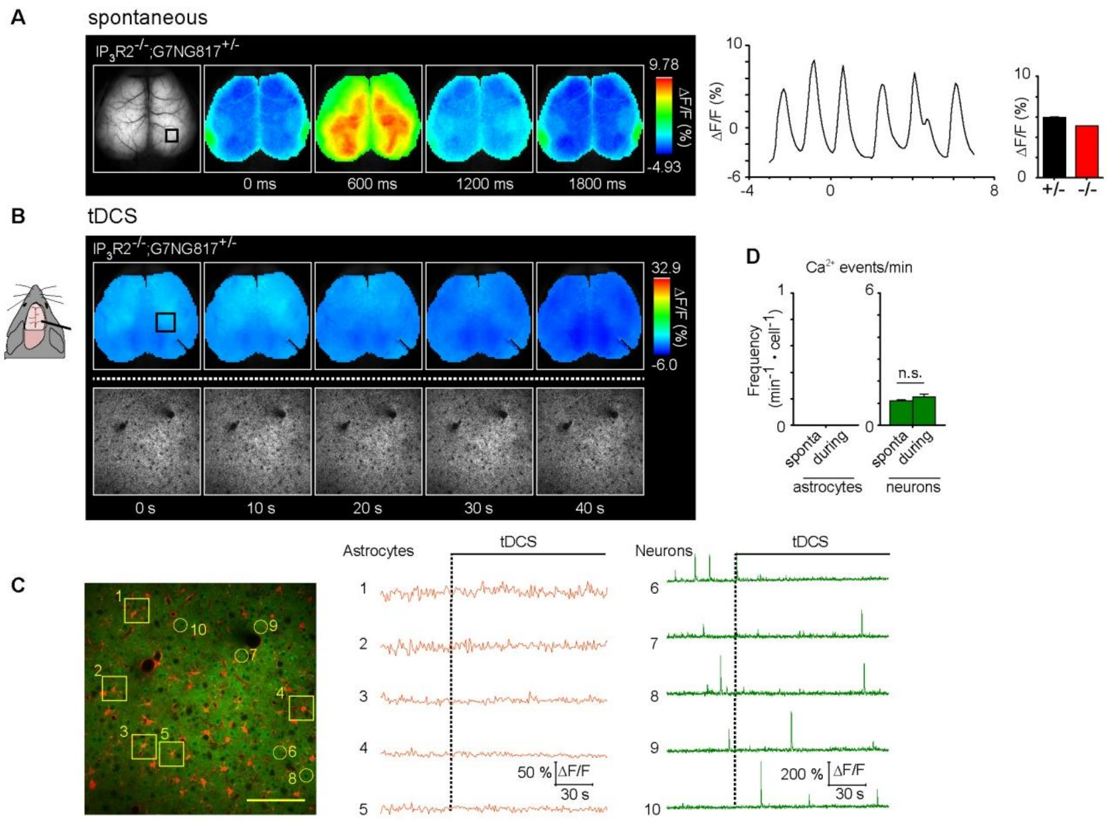

Supplementary Figure 7. tDCS does not induce Ca^{2+} surges in IP_3R2 KO mice.

A. Slow oscillations are observed in a urethane-anesthetized IP_3R2 knockout mouse which carries the G7NG817 transgene ($IP_3R2^{-/-}$;$G7NG817^{+/-}$). The G-CaMP7 signal of the area marked with a square is plotted on the right. The amplitude of the slow oscillations is similar between $IP_3R2^{-/-}$;$G7NG817^{+/-}$ and $IP_3R2^{+/-}$;$G7NG817^{+/-}$ (N = 6 and 3, respectively)

B. tDCS is applied to a urethane-anesthetized G7NG817 transgene ($IP_3R2^{-/-}$;$G7NG817^{+/-}$). Although the tDCS parameter is the same as in Figure 1D, Ca^{2+} surges were not induced in the $IP_3R2^{-/-}$;$G7NG817^{+/-}$ mouse.

C. Intracranial two-photon imaging was performed in a urethane-anesthetized $IP_3R2^{-/-}$;$G7NG817^{+/-}$ mice with the glia maker SR101 (red). Cells marked with circles and squares point to neurons and astrocytes, respectively. G-CaMP7 signals of these cells before and during tDCS are plotted on the bottom. Scale bar: 100 µm.

D. Data from three urethane-anesthetized $IP_3R2^{-/-}$;$G7NG817^{+/-}$ mice show that tDCS did not induce Ca^{2+} surges in astrocytes during tDCS under urethane anesthesia.

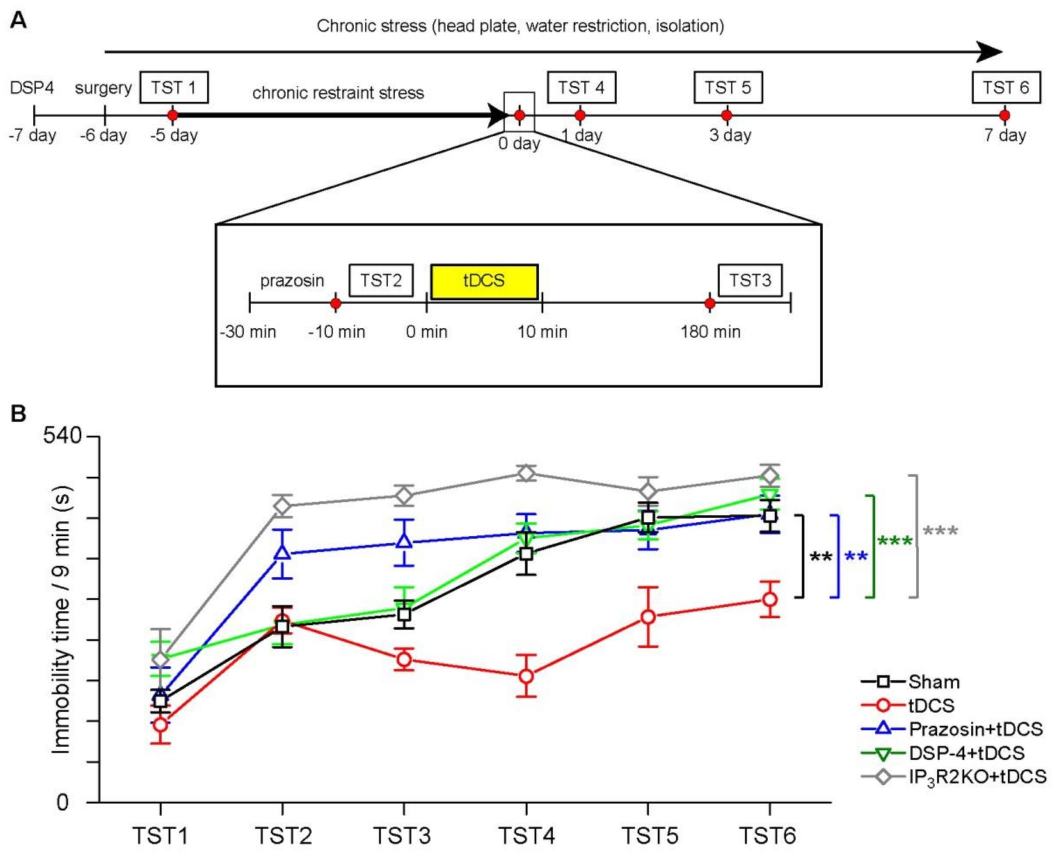

Supplementary Figure 8. tDCS alleviates a mouse model of depression by chronic restraint stress
A. Schematic diagram of the schedule for the behavioral test. TST: tail suspension test.
B. tDCS reduces depression-like behavior after 1 week. TST one week after tDCS (TST6) shows that tDCS group (N = 10) are more active than the group without tDCS treatment (sham; N = 10). This effect is blocked by acute prazosin administration 30 min before tDCS (N = 10) and DSP-4 treatment (N = 9), as well as in IP3R2 KO mice (N = 9).

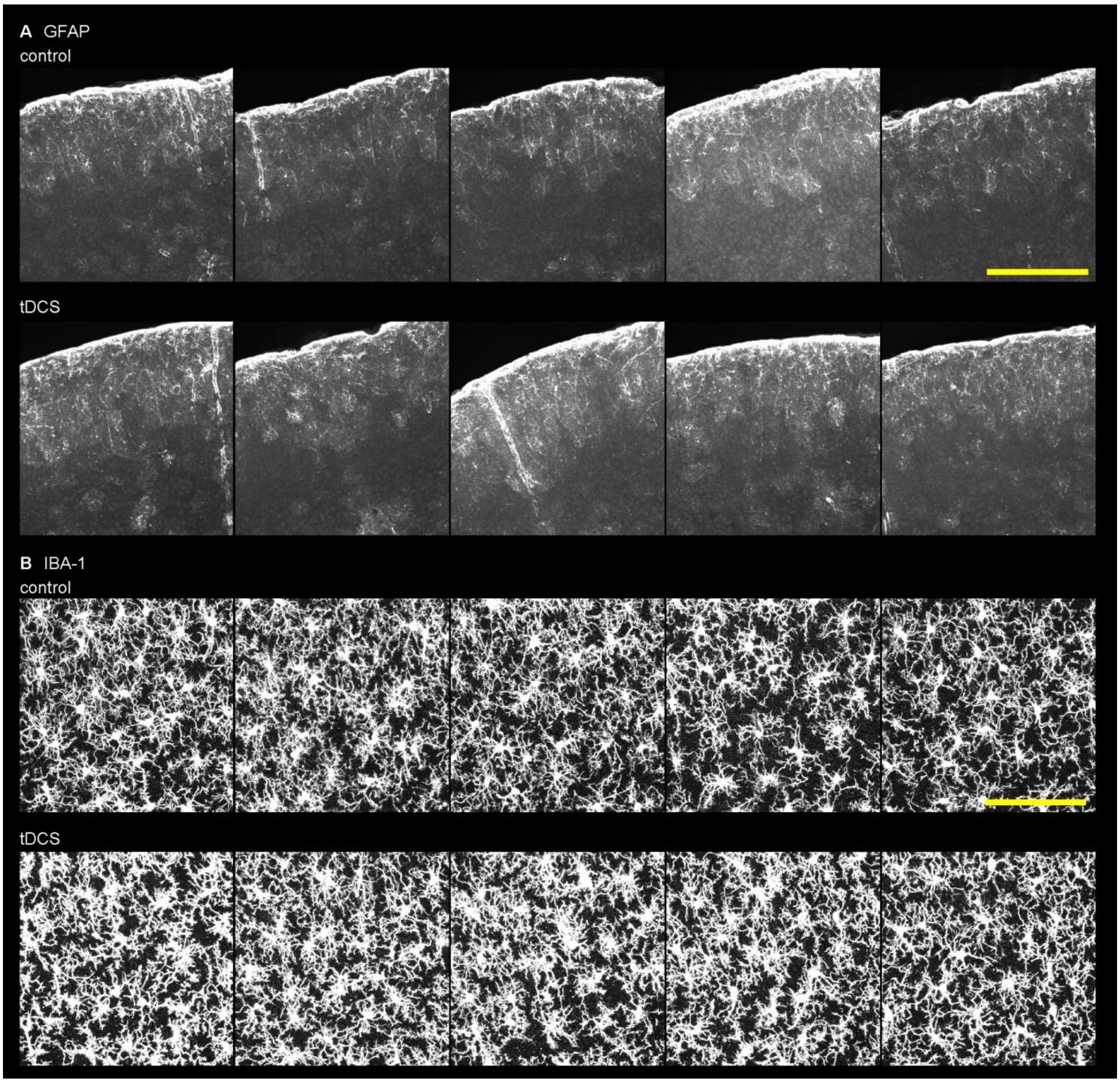

Supplementary Figure 9. Cortical GFAP and IBA-1 staining patterns at the anodal site three hours after tDCS

Five sham-operated adult C57BL/6 mice are compared with five mice that underwent tDCS (0.1 mA, 10 minutes, anodal site: above the primary visual cortex). The mice were sacrificed 3 hours after the offset of tDCS and immunohistochemistry was performed for GFAP and IBA-1 to examine the reactivity of astrocytes and microglia, respectively. A. GFAP immunostaining of the superficial layers of the primary visual cortex in control and tDCS conditions. B. Layer 2/3 IBA-1 immunostaining in the same set of mice as a. The images are the maximum intensity projections of 50 µm stacked images. Neither GFAP nor IBA-1 staining shows obvious inflammatory patterns.
Scale bar: A, 300 µm; B, 100µm

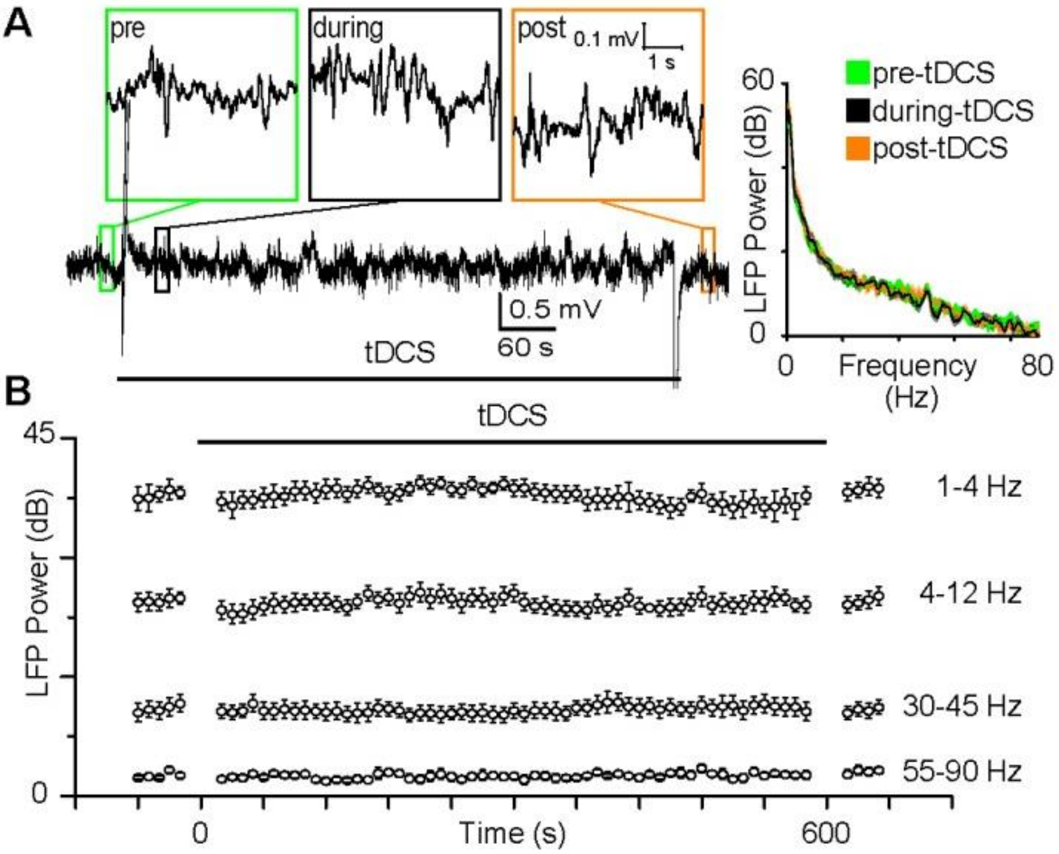

Supplementary Figure 10. Cortical LFP patterns do not show obvious alternations with tDCS

A. Cortical LFP traces from a urethane-anesthetized C57BL/6 mouse for a tDCS session. The insets are magnified views of pre- ([-20 s, -15 s], where 0 s is the onset of tDCS), during- ([40 s, 45 s]), and post-tDCS ([640 s, 645 s]) periods. Mean spectra from 8 anesthetized mice are plotted on the right. There is no obvious spectral difference in the field potential patterns.

B. Time plots of cortical LFP powers of various frequencies, showing that tDCS does not have an obvious impact on the LFP pattern.

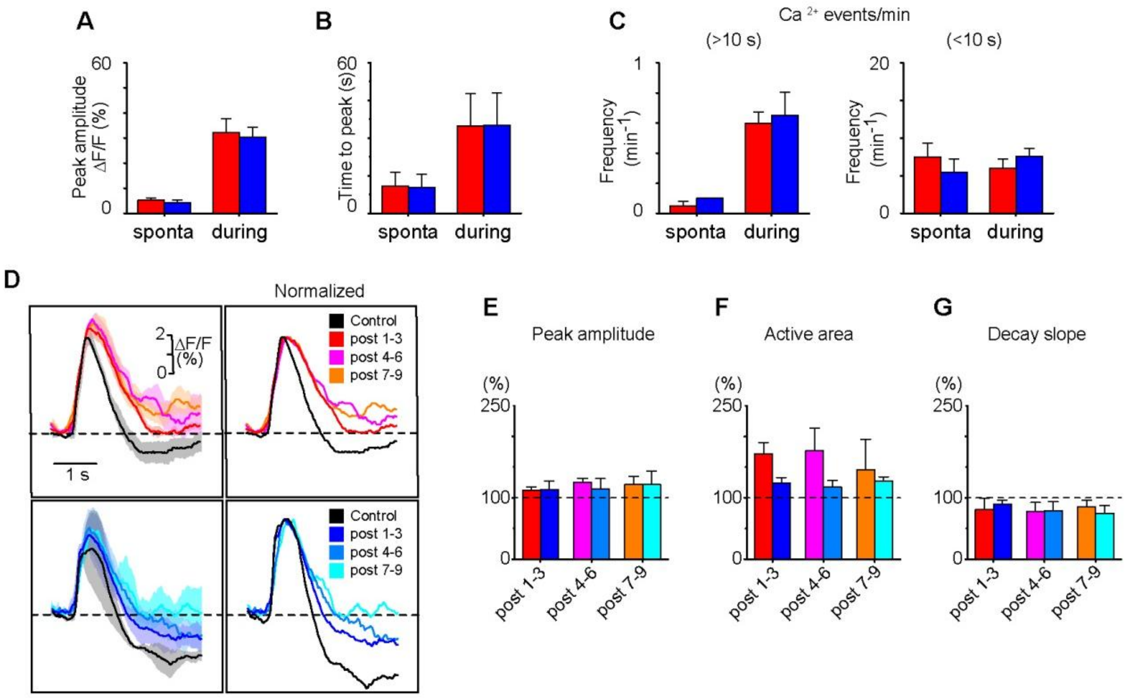

Supplementary Figure 11. Demonstration of tDCS-induced sensory plasticity in heterozygous G7NG817

The tDCS experiments presented in Figure 4C-F are replicated in G7NG817 heterozygous mice. tDCS in heterozygous G7NG817 also induces visual-evoked Ca^{2+} response (D; N = 4 mice). Similar to the homozygous mouse, tDCS induced an enhancement in the visual-evoked Ca^{2+} response amplitude (E), active area (F), and a decline in the decay slope (G)

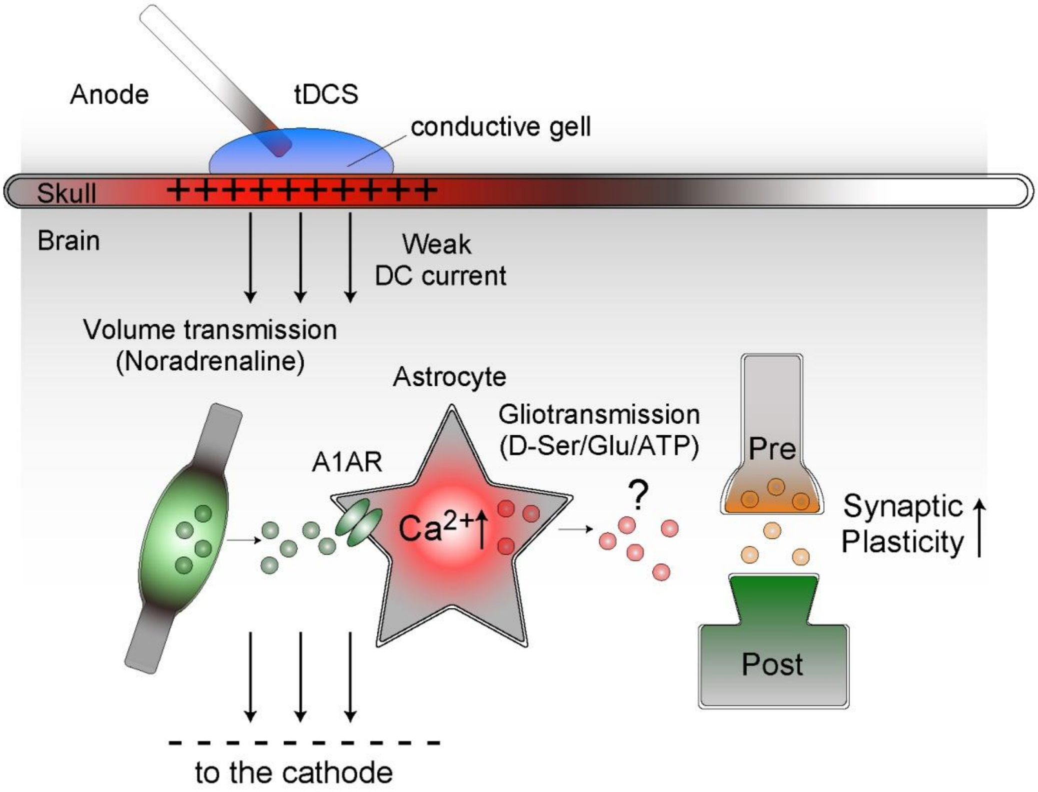

Supplementary Figure 12. Schematic diagram for tDCS-induced Ca^{2+} elevations in the cortex. See discussions in the main text.
